# Development of a protocol on safe practices for insulin preparation and administration

**DOI:** 10.1590/0034-7167-2025-0345

**Published:** 2026-05-08

**Authors:** Tatiana Rebouças Moreira, Rhanna Emanuela Fontenele Lima de Carvalho, Consuelo Helena Aires de Freitas, Daniel Duarte Gadelha, Adriana Catarina de Souza Oliveira, Lucilane Maria Sales Silva

**Affiliations:** I Universidade Estadual do Ceará. Fortaleza, Ceará, Brazil; II Universidade Federal do Ceará. Fortaleza, Ceará, Brazil; III Universidad Católica de Murcia. Múrcia, Região de Múrcia, Spain

**Keywords:** Health Personnel, Diabetes *Mellitus*, Insulin, Good Manipulation Practices, Guideline., Pessoal de Saúde, Diabetes *Mellitus*, Insulina, Boas Práticas de Manipulação, Guia., Personal de Salud, Diabetes *Mellitus*, Insulina, Buenas Prácticas de Manipulación, Guía.

## Abstract

**Objectives:**

to develop and validate a protocol for safe insulin preparation and administration in healthcare settings.

**Methods:**

this methodological study, conducted from September 2022 to May 2024, had two phases. Phase one involved protocol development and validity by 46 experts using AGREE II for content and methodological aspects. Phase two trained 40 healthcare professionals in Fortaleza, Brazil, with pre/post-tests assessing knowledge acquisition. Data were analyzed using descriptive statistics, reproducibility measures (Cronbach’s alpha, McDonald’s omega), Content Validity Index, and various statistical tests.

**Results:**

the protocol achieved high quality and internal consistency, with a Content Validity Index of 97.83-100 (p<0.001). Its overall quality score was 95.7% (6.70 ± 0.47), and all six domains scored ≥ 90.0. High reproducibility was confirmed (alpha=0.94, omega=0.95). After training, correct responses significantly increased from 32% to 70%.

**Conclusions:**

the validated protocol is a high-quality technology that significantly enhances healthcare professionals’ knowledge of safe insulin practices.

## RESUMO

**Objetivos:**

desenvolver e validar protocolo para preparo/administração segura de insulina em ambientes de saúde.

**Métodos:**

estudo metodológico, conduzido de setembro/2022 a maio/2024, em duas fases. Fase um envolveu desenvolvimento/validação do protocolo por 46 especialistas, utilizando AGREE II para aspectos de conteúdo/metodológicos. Fase dois treinou 40 profissionais de saúde em Fortaleza, Brasil, com pré/pós-testes para avaliar aquisição de conhecimento. Dados foram analisados através de estatística descritiva, medidas de reprodutibilidade (alfa de Cronbach, ômega de McDonald), Índice de Validade de Conteúdo e testes estatísticos.

**Resultados:**

o protocolo alcançou alta qualidade e consistência interna, com Índice de Validade de Conteúdo de 97,83-100 (p<0,001). Seu escore geral de qualidade foi de 95,7% (6,70±0,47), e todos os seis domínios obtiveram pontuação≥90,0. Confirmou-se alta reprodutibilidade (alfa=0,94; ômega=0,95). Após treinamento, as respostas corretas aumentaram de 32% para 70%.

**Conclusões:**

o protocolo validado representa tecnologia de alta qualidade que aprimora significativamente o conhecimento dos profissionais sobre práticas seguras de insulina.

## RESUMEN

**Objetivos:**

desarrollar y validar protocolo para la preparación/administración segura de insulina en entornos sanitarios.

**Métodos:**

estudio metodológico, realizado entre septiembre/2022 y mayo/2024, en fases. Fase uno consistió en el desarrollo/validación del protocolo por parte de 46 expertos, utilizando el AGREE II para su contenido y aspectos metodológicos. Fase dos capacitó a 40 profesionales en Fortaleza, Brasil, con pruebas previas/posteriores para evaluar la adquisición de conocimientos. Se analizaron datos mediante estadística descriptiva, medidas de reproducibilidad (alfa de Cronbach, omega de McDonald), Índice de Validez de Contenido y pruebas estadísticas.

**Resultados:**

el protocolo alcanzó alta calidad y consistencia interna, con Índice de Validez de Contenido de 97,83-100 (p<0,001). Su puntuación de calidad general fue del 95,7% (6,70±0,47), y los seis dominios obtuvieron una puntuación≥90,0. Se confirmó alta reproducibilidad (alfa=0,94; omega=0,95). Tras el entrenamiento, las respuestas correctas aumentaron del 32% al 70%.

**Conclusiones:**

el protocolo validado constituye tecnología de alta calidad que mejora significativamente el conocimiento de profesionales sobre prácticas seguras de insulina.

## INTRODUCTION

In the pharmacological management of diabetes mellitus (DM), insulin therapy plays a key role. The Institute for Safe Medication Practices classifies insulin as a high-alert medication (HAM), meaning it carries a heightened risk of significant harm due to potential administration errors^(1)^.

Despite this risk, HAMs are widely used in both hospital and outpatient settings for various clinical conditions, highlighting the need for continuous education of healthcare professionals and the implementation of surveillance systems to prevent errors and adverse events. Consequently, HAMs should be a priority in medication error prevention programs^(2)^.

Insulin is among the top five drugs associated with harm in adult and pediatric patients due to administration errors. As a narrow therapeutic index medication, excessive doses can lead to severe complications such as hypoglycemia, encephalopathy, pulmonary edema, liver damage, hypoglycemic coma, and death^(3)^.

Errors in insulin preparation and administration are frequently reported by healthcare professionals, compromising patient safety. These include incorrect dosing due to abbreviations and symbols in prescriptions, miscalculated doses from using syringes graduated in milliliters (ml) instead of international units, administration of insulins with pharmacokinetic profiles different from those prescribed (e.g., rapid-acting instead of basal insulins), improper timing between insulin administration and meals (causing glycemic fluctuations), lack of homogenization of suspended insulins, incorrect infusion pump programming in inpatient settings, among other^(4)^.

To mitigate these risks, continuing health education and institutional protocols for safe medication preparation and administration are essential. These measures help healthcare teams acknowledge prescription and administration risks, identify drug incompatibilities, and adhere to therapeutic guidelines regarding dosage, administration routes, timing, reconstitution, dilution, storage, and adverse effects^(5)^.

Care protocols and performance indicators should guide all stages of medication management according to predefined criteria and responsibilities^(6)^. Their advantages include enhanced patient and professional safety, care standardization, improved decision-making, easier adoption of new technologies, innovation in clinical practice, optimized resource allocation, and greater transparency and cost control. Additionally, protocols support the development of process and outcome indicators, knowledge dissemination, professional communication, care coordination, standardized procedures, and improved workflow understanding in medication use^(7,8)^.

Patient safety depends on how medications are prescribed, dispensed, administered, and monitored. The more prepared a healthcare service is to prevent errors, the safer the patient will be. Establishing medication error assessment and prevention programs across healthcare institutions is crucial, and professionals must be continuously trained for safe care monitoring and execution^(2)^.

## OBJECTIVES

To develop and validate a protocol for safe insulin preparation and administration in healthcare settings.

## METHODS

### Ethical aspects

The study adhered to ethical principles and guidelines for human research, complying with international standards. Approvals were granted by the Research Ethics Committees of *Universidade Estadual do Ceará* (Brazil) and *Hospital Universitário Walter Cantídio* (Brazil).

### Study design, period and site

A methodological study was conducted from August 2023 to May 2024 in two sequential phases. The first phase involved the protocol development and validity by 46 expert judges, considering the document’s content and methodological aspects using the Appraisal of Guidelines for Research & Evaluation II (AGREE II). The second phase was quasi-experimental, involving training on the protocol with 40 healthcare professionals from a referral hospital in Fortaleza, Brazil, with preand post-tests applied to compare knowledge before and after training. Both samples were selected through convenience sampling, and no sample size was determined, since the aim was to recruit as many participants as possible.

### Sample, inclusion and exclusion criteria

The experts involved in the validity process were professionals skilled in diabetes care, with practical experience, and selected based on Jasper’s criteria. An expert must meet at least two of the following criteria: experience-based knowledge/skills; specialized authority in the field; expertise in specific studies; passing a test to identify experts; and high rating by an authority.

### Study protocols

The protocol was developed using the methodological framework by Pimenta *et al*.^(9)^ as a facilitating method for creating technology that meets the target audience’s needs and objectives, following the stages as follows: origin; objective; development group; conflict of interest; evidence; review; flowchart; indicators; validity by the professionals who will use the protocol; limitations; and implementation plan.

For content assessment and methodological quality, a validated version of AGREE II was used, consisting of 23 items organized into six domains: scope and purpose (overall objectives, health questions covered, and population); stakeholder involvement (development team, opinions and preferences, target users); rigor of development (methods for evidence search, criteria for evidence selection, strengths and limitations of the evidence, methods for formulating recommendations, benefits, side effects, and risks, link between recommendations and supporting evidence, expert review, updates); clarity of presentation (specific and unambiguous recommendations, clearly presented options for addressing the health issue, key recommendations); applicability (facilitators and barriers, advice for practice, resources required for application of the recommendations, monitoring); editorial independence (lack of influence over the guideline content and conflicts of interest); the overall guideline assessment; and two overall rating items^(10)^.

The quasi-experimental phase took place in person, utilizing a pre-test instrument based on the protocol’s content to assess prior knowledge before the training process. A post-test instrument (identical to the pre-test) was administered afterward to assess participants’ knowledge gain following the protocol training.

The preand post-tests contained 12 multiple-choice questions on the following topics, as covered in the protocol on safe practices for insulin preparation and administration: storage; validity; transport; pharmacokinetic classification; administration devices; combined use of insulin; insulin absorption rate; hypoglycemia; intravenous insulin use; continuous subcutaneous insulin infusion system; diabetes-related waste management and disposal; and safe practices for insulin preparation and administration.

Four training sessions on the protocol were conducted, each lasting approximately 1 hour and 40 minutes, including 20 minutes for the pre-test, 1 hour for theoretical and practical explanation, and 20 minutes for the post-test, involving 40 professionals. Professionals involved in the healthcare for patients with diabetes for more than six months were included. Professionals who were absent during the data collection period were excluded. The aim was to assess knowledge improvement on the topic after training.

### Data analysis and statistics

The data were processed using the Statistical Package for the Social Sciences (SPSS) version 29.0. Descriptive statistics were analyzed, as well as the exact binomial test to assess the content items of the created technology. Reproducibility was analyzed using Cronbach’s alpha and McDonald’s omega, as well as the Content Validity Index (CVI). Statistical tests were performed to characterize variables, assess protocol quality, and assess knowledge improvement between the preand post-tests in paired samples. A significance level of p < 0.05 was considered.

## RESULTS

**Figure 1:**
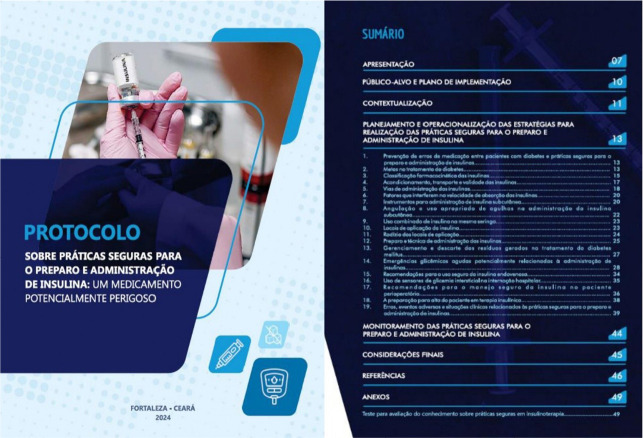
Cover and summary of the protocol on safe practices for insulin preparation and administration, Fortaleza, Ceará, Brazil, 2024

Following an extensive literature review^(5)^ and a situational analysis of errors reported by healthcare professionals in insulin preparation and administration, a protocol was developed (Figure 1). This protocol compiles key theoretical aspects and practical examples relevant to managing individuals with diabetes using insulin in healthcare settings.

The validity process involved 86 healthcare professionals, including nurses, physicians, nutritionists, physiotherapists, and a psychologist. Of these, 46 expert judges participated in content validity, while 40 healthcare professionals were part of the quasi-experimental phase. Comparative analysis revealed significant differences between judges and healthcare professionals, with the former having higher age, professional experience, and educational qualifications (Mann-Whitney test, p < 0.001; Fisher’s exact test, p < 0.001, Table 1).

**Table 1:** Characterization of healthcare professionals and judges, Fortaleza, Ceará, Brazil, 2024

Characterization of healthcare professionals and judges	Total (%)	Healthcare professionals N=40^1^ (%)	Judges N=46^1^ (%)	*p* value^2^
Age	33.5 ± 8	28 ± 4	38 ± 12	<0.001
Length of professional experience	8.9 ± 6.8	3.5 ± 3.2	13.7 ± 10.3	<0.001
Time working in the area	5.7 ± 4.9	2.1 ± 2.9	8.8 ± 6.9	<0.001
Sex Female Male	74 (86) 12 (14)	32 (80) 8 (9)	42 (91) 4 (5)	0.31
Profession Nursing Nutrition Physiotherapy Medicine Psychology	45 (52) 15 (17) 10 (%) 15 (17) 1 (1)	19 (47.5) 8 (20) 4 (10) 9 (22.5) 0 (0)	26 (57) 7 (15) 6 (13) 6 (13) 1 (2)	0.6447
Level of education Undergraduate degree Specialization Master’s degree PhD	15 (17.4) 41 (47.6) 30 (34.8) 11 (12.7)	15 (37.5) 24 (60) 5 (12.5) 0 (0)	0 17 (37) 25 (54) 11 (24)	<0.001

*1Mean ± Standard Deviation; n (%); 2Mann-Whitney test; chi-square test of independence; Fisher’s exact test.*

Assessment of the protocol using AGREE II demonstrated domain scores above 90% with a high CVI. Statistical significance was observed across all domains and items (p < 0.001; Table 2).

**Table 2:** Protocol quality score according to Appraisal of Guidelines for Research & Evaluation II items, Fortaleza, Ceará, Brazil, 2024

AGREE II item	N score = 461	Centesimal score^1^	CVI	*p* value^2^
AGREE 1	6.76 ± 0.52 (7.00)	96.57 ± 0.074	100.00	<0.001
AGREE 2	6.72 ± 0.54 (7.00)	96.00 ± 0.077	100.00	<0.001
AGREE 3	6.74 ± 0.53 (7.00)	96.28 ± 0.075	100.00	<0.001
DOM1: Scope and purpose	95.7 ± 7.4 (100.0)	-	100.00	<0.001
AGREE 4	6.28 ± 0.86 (7.00)	89.71 ± 0.122	97.83	<0.001
AGREE 5	6.22 ± 0.87 (6.00)	88.85 ± 0.124	95.65	<0.001
AGREE 6	6.72 ± 0.54 (7.00)	96.00 ± 0.077	100.00	<0.001
DOM 2: Stakeholder involvement	90 ± 9 (89)	-	96.49	<0.001
AGREE 7	6.87 ± 0.34 (7.00)	98.14 ± 0.048	100.00	<0.001
AGREE 8	6.67 ± 0.60 (7.00)	95.28 ± 0.085	100.00	<0.001
AGREE 9	6.50 ± 0.75 (7.00)	92.85 ± 0.107	97.83	<0.001
AGREE 10	6.61 ± 0.68 (7.00)	94.42 ± 0.097	100.00	<0.001
AGREE 11	6.57 ± 0.69 (7.00)	93.85 ± 0.098	97.83	<0.001
AGREE 12	6.52 ± 0.66 (7.00)	93.14 ± 0.094	100.00	<0.001
AGREE 13	6.78 ± 0.42 (7.00)	96.85 ± 0.006	100.00	<0.001
AGREE 14	6.61 ± 0.71 (7.00)	94.42 ± 0.101	97.83	<0.001
DOM 3: Rigor of development	94.0 ± 7.1 (95.8)	-	99.18	<0.001
AGREE 15	6.74 ± 0.53 (7.00)	96.28 ± 0.075	100.00	<0.001
AGREE 16	6.70 ± 0.55 (7.00)	95.71 ± 0.078	100.00	<0.001
AGREE 17	6.67 ± 0.60 (7.00)	95.28 ± 0.085	100.00	<0.001
DOM 4: Clarity of presentation	95 ± 8 (100)	-	100.00	<0.001
AGREE 18	6.50 ± 0.75 (7.00)	92.85 ± 0.107	97.83	<0.001
AGREE 19	6.74 ± 0.49 (7.00)	96.28 ± 0.070	100.00	<0.001
AGREE 20	6.61 ± 0.65 (7.00)	96.28 ± 0.092	100.00	<0.001
AGREE 21	6.63 ± 0.57 (7.00)	94.71 ± 0.081	100.00	<0.001
DOM 5: Applicability	93.7 ± 7.9 (95.8)	-	99.45	<0.001
AGREE 22	6.76 ± 0.60 (7.00)	96.57 ± 0.085	97.83	<0.001
AGREE 23	6.70 ± 0.63 (7.00)	95.71 ± 0.090	97.83	<0.001
DOM 6: Editorial independence	95.5 ± 9.9 (100.0)	-	97.83	<0.001
**OVERALL QUALITY**	6.70 ± 0.47 (7.00)	95.71 ± 0.067	100.00	<0.001

*1Mean ± standard deviation (median); n (%); 2Binomial test.*

All 46 judges recommended the protocol for use, with 80% approving the initial version and the remaining 20% suggesting minor adjustments that were incorporated. Furthermore, all 23 AGREE II items and the overall quality assessment showed over 80% agreement, indicating high reliability.

**Figure 2:**
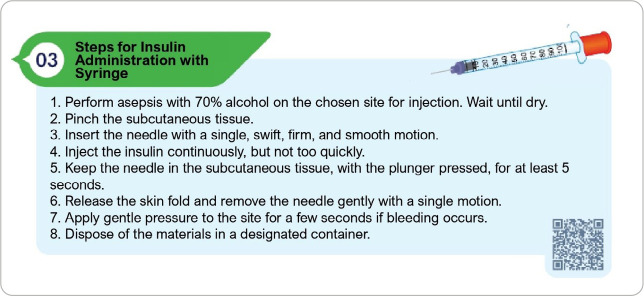
Example of modifications made, according to judges’ recommendations, for the construction of the final version of the protocol, Fortaleza, Ceará, Brazil, 2024

Judges recommended adjustments such as illustration refinements, spelling corrections, replacing “diabetic” with “person with diabetes” (as per Speight *et al*.^(11)^), and adding a QR code for a step-by-step demonstration of insulin preparation and administration. These updates were integrated into the final protocol, as illustrated in Figure 2.

During the quasi-experimental phase, preand post-tests assessed knowledge improvement following training on safe insulin practices. Correct responses increased significantly from 32% (152 responses) in the pre-test to 70% (338 responses) in the post-test (McNemar test with continuity correction, p < 0.05). An absolute improvement was observed across all questionnaire items, with statistical significance in 11 out of 12 items (Table 3).

**Table 3:** Increased knowledge about safe practices for preparing and administering insulin after protocol training, Fortaleza, Ceará, Brazil, 2024

Questionnaire variables	N	Pre, N = 401	Post, N = 401	*p* value^1^
Packaging	80	23 (58)	37 (93)	<0.001
Validity	80	7 (18)	28 (70)	<0.001
Transport	80	10 (25)	35 (88)	<0.001
Pharmacokinetic classification	80	8 (20)	28 (70)	<0.001
Administration devices	80	9 (23)	27 (68)	<0.001
Combined use of insulin	80	12 (30)	25 (63)	0.006
Insulin absorption rate	80	12 (30)	23 (58)	0.029
Hypoglycemia	80	24 (60)	28 (70)	0.453
Intravenous insulin use	80	12 (30)	28 (70)	<0.001
Continuous subcutaneous insulin infusion system	80	9 (23)	22 (55)	0.006
Diabetes-related waste management and disposal	80	6 (15)	25 (63)	<0.001
Safe practices for insulin preparation and administration	80	20 (50)	32 (80)	0.006
Total	960	152 (32)	338 (70)	<0.001

*1Binomial test.*

## DISCUSSION

In the context of patient safety, the validity of healthcare technologies is crucial for ensuring the quality of developed products. This study developed and validated a protocol on safe practices for insulin preparation and administration in healthcare settings. This effort contributes to implementing models for preventing errors related to the use of this medication, emphasizing its classification as a HAM and the harmful impact resulting from its misuse.

To achieve excellence in healthcare technologies, the importance of methodological studies stands out. These studies involve the validity processes of models and research methods, focusing on methodological development and the quality of the resulting products^(12)^.

In healthcare settings, particularly in hospital admissions, due to the benefits of insulin treatment in managing DM, patients are highly likely to be treated with insulin alone or in combination with oral antidiabetics. However, errors related to insulin administration have been alarmingly reported, frequently causing harm to patients^(13)^.

The most common types of errors in insulin administration include incorrect, omitted, or delayed doses. Common contributing factors to insulin errors include transcription errors, medication calculation errors, non-adherence to protocols, and communication failures^(14)^.

It is argued that insulin administration incidents are largely preventable if risk reduction strategies are implemented, such as: establishing interdisciplinary team workflows supported by management units; standardizing digitalized prescriptions and computerized systems; avoiding acronyms and abbreviations; performing double-checks; adhering to policies; and reinforcing the implementation of specific protocols on the safe use of insulin tied to the continuing education of healthcare teams^(15)^.

Beyond the quality of records, secure communication is another crucial aspect in healthcare settings, particularly during critical moments such as shift changes, transitions of care (e.g., admission, transfer, discharge), and effective communication for educating individuals with diabetes^(16)^.

Another pillar of effective communication is the healthcare team’s commitment to using assertive, empathetic, motivating, inclusive, and respectful language, fostering shared decision-making, effective diabetes education, and aiming to establish bonds, encourage autonomy, and empower self-care management in a horizontal relationship between educator and learner^(11)^.

Therefore, healthcare professionals’ approach towards individuals with diabetes is crucial for engagement in self-care, emotional balance, and overall well-being. Imposing guidance and treatments through threats of future complications is an inappropriate and counterproductive choice that instills fear and unnecessary panic, creating a negative stigma around the condition. Hence, a sensitive and individualized educational approach should underpin the conduct of healthcare professionals working with individuals with diabetes^(17)^.

Regarding safe practices for insulin preparation and administration, critical points in healthcare settings and in the educational process for individuals with diabetes deserve greater attention and emphasis in institutional protocols and continuing education programs. These points include insulin types and pharmacokinetics (inadequate interval between administration and meals), storage, rotation, appropriate administration devices, technique, and disposal and management of sharps and other waste resulting from insulin therapy. Each of these aspects should be carefully discussed with the entire healthcare team and the support network for individuals with diabetes^(18,19)^.

A study aimed at analyzing the relationship between health education and adherence to self-care actions by individuals with diabetes revealed an improvement in self-care actions related to safe insulin therapy practices following diabetes education during nursing consultations, highlighting the effectiveness of these interventions in promoting safe insulin therapy^(20)^.

Regarding continuing education, the present study demonstrated a significant increase in healthcare professionals‘ knowledge about safe practices for insulin preparation and administration after training on the protocol, underscoring the importance of adhering to these practices in healthcare settings.

These findings are supported by evidence supporting the idea that team training is a factor directly related to identifying errors and establishing strategies for standardizing practices to prevent new errors, as well as events related to patient safety risks and the quality of care for individuals with diabetes^(2)^.

In the context of diabetes education, special attention should be given to vulnerable populations more prone to errors in safe insulin therapy practices, such as older adults, who may experience physiological changes that impair their ability to follow the stages for preparation and administration, including reduced visual acuity. Children and pregnant women also require special consideration, as their unique needs, such as low doses and insulin use only during pregnancy, respectively, increase their risk of incidents. This highlights the importance of supervised care and continuous support networks^(15)^.

A study assessing the effectiveness of continuing education in acquiring knowledge among professionals about glycemic monitoring and hypoglycemia management showed that the intervention was effective in enhancing diabetes knowledge, facilitating the standardization of healthcare practices and ensuring safe care for individuals with diabetes^(21)^.

Furthermore, in the realm of continuing diabetes education, the importance of fostering strategies for monitoring indicators is emphasized as a pillar for planning actions and programs aimed at quality care, allowing for the redirection and planning of actions, interventions, and health updates^(6)^.

### Study limitations

This study presented some limitations, such as the lack of a quasi-experimental phase in different institutions due to the need to complete the research. Conducting the quasi-experimental phase in only one reference hospital for DM care may have implied a higher baseline knowledge about the protocol’s topic among participating professionals compared to the general healthcare workforce, potentially resulting in a greater knowledge increase post-training.

### Contributions to nursing, health, or public policy

The findings from this research reinforce the critical need for strategies that promote safety in insulin preparation and administration. Protocol validity and implementation are presented as essential tools for error prevention, which in turn elevates the quality of care and safety for patients with diabetes by standardizing evidence-based practices.

## CONCLUSIONS

The protocol was considered a valid technology, with a high overall quality score, contributing to increased healthcare professionals’ knowledge on safe practices for insulin preparation and administration in healthcare settings. The findings of this study can drive improvements in diabetes care, build on previous research, and guide future investigations to meet expected outcomes.

## Data Availability

The research data are available within the article.
